# Prevalence, Symptom Burden, and Underdiagnosis of Chronic Obstructive Pulmonary Disease in a Lung Cancer Screening Cohort

**DOI:** 10.1513/AnnalsATS.201911-857OC

**Published:** 2020-07

**Authors:** Mamta Ruparel, Samantha L. Quaife, Jennifer L. Dickson, Carolyn Horst, Sophie Tisi, Helen Hall, Magali N. Taylor, Asia Ahmed, Penny J. Shaw, Stephen Burke, May-Jan Soo, Arjun Nair, Anand Devaraj, Karen Sennett, John R. Hurst, Stephen W. Duffy, Neal Navani, Angshu Bhowmik, David R. Baldwin, Sam M. Janes

**Affiliations:** ^1^ Lungs for Living Research Centre, University College London (UCL) Respiratory; ^2^ Research Department of Behavioural Science and Health; ^7^ UCL Centre for Inflammation and Repair, University College London, London, United Kingdom; ^3^ Department of Radiology and; ^9^ Department of Thoracic Medicine, University College London Hospital, London, United Kingdom; ^4^ Department of Radiology and; ^10^ Department of Thoracic Medicine, Homerton University Hospital, London, United Kingdom; ^5^ Department of Radiology, Royal Brompton Hospital, London, United Kingdom; ^6^ Killick Street Health Centre, London, United Kingdom; ^8^ Wolfson Institute of Preventive Medicine, Barts and The London School of Medicine and Dentistry, Queen Mary University, London, United Kingdom; and; ^11^ Respiratory Medicine Unit, David Evans Research Centre, Nottingham University Hospitals, Nottingham, United Kingdom

**Keywords:** lung cancer screening, low-dose computed tomography, emphysema, chronic obstructive pulmonary disease, case finding

## Abstract

**Rationale:** Individuals eligible for lung cancer screening (LCS) by low-dose computed tomography (LDCT) are also at risk of chronic obstructive pulmonary disease (COPD) due to age and smoking exposure. Whether the LCS episode is useful for early detection of COPD is not well established.

**Objectives:** To explore associations between symptoms, comorbidities, spirometry, and emphysema in participants enrolled in the Lung Screen Uptake Trial.

**Methods:** This cross-sectional study was a prespecified analysis nested within Lung Screen Uptake Trial, which was a randomized study testing the impact of differing invitation materials on attendance of 60- to 75-year-old smokers and ex-smokers to a “lung health check” between November 2015 and July 2017. Participants with a smoking history ≥30 pack-years and who quit ≤15 years ago, or meeting a lung cancer risk of ≥1.51% via the Prostate Lung Colorectal Ovarian model or ≥2.5% via the Liverpool Lung Project model, were offered LDCT. COPD was defined and classified according to the GOLD (Global Initiative for Obstructive Lung Disease) criteria using prebronchodilator spirometry. Analyses included the use of descriptive statistics, chi-square tests to examine group differences, and univariable and multivariable logistic regression to explore associations between symptom prevalence, airflow limitation, and visually graded emphysema.

**Results:** A total of 560 of 986 individuals included in the analysis (57%) had prebronchodilator spirometry consistent with COPD; 67% did not have a prior history of COPD and were termed “undiagnosed.” Emphysema prevalence in those with known and “undiagnosed” COPD was 73% and 68%, respectively. A total of 32% of those with “undiagnosed COPD” had no emphysema on LDCT. Inhaler use and symptoms were more common in the “known” than the “undiagnosed” COPD group (63% vs. 33% with persistent cough [*P* < 0.001]; 73% vs. 33% with dyspnea [*P* < 0.001]). Comorbidities were common in all groups. Adjusted odds ratio (aOR) of respiratory symptoms were more significant for airflow obstruction (aOR GOLD 1 and 2, 1.57; confidence interval [CI], 1.14–2.17; aOR GOLD 3 and 4, 4.6; CI, 2.17–9.77) than emphysema (aOR mild, 1.12; CI, 0.81–1.55; aOR moderate, 1.33; CI, 0.85–2.09; aOR severe, 4.00; CI, 1.57–10.2).

**Conclusions:** There is high burden of “undiagnosed COPD” and emphysema in LCS participants. Adding spirometry findings to the LDCT enhances identification of individuals with COPD.

Clinical trial registered with www.clinicaltrials.gov (NCT02558101).

Chronic obstructive pulmonary disease (COPD) is the third most common cause of death globally, after coronary heart disease (CHD) and stroke (1). The presence of emphysema, airflow limitation, increasing spirometric COPD severity, and exacerbation frequency are all associated with a greater risk of lung cancer (2, 3), though this effect is reduced after adjustment for smoking history and other confounders (4). Biologically, this association may be explained by a combination of chronic inflammation, impaired mucocilliary action, DNA damage and aberrant repair, and genetic susceptibility (5).

Lung cancer screening (LCS) using low-dose computed tomography (LDCT) significantly reduced the relative risk of lung cancer-specific and all-cause mortality by 20% and 6.7%, respectively, in the National Lung Screening Trial (NLST) (6). Both the Dutch–Belgian LCS trial and the Multicentric Italian Lung Detection study provide further evidence in a European population, in support of this finding (7, 8). Evidence suggests cohorts at higher risk for lung cancer may be more likely to benefit from LCS than those with lower risk (9), with a twofold increase in lung cancer prevalence, greater stage shift, and reduced overdiagnosis among those with airflow limitation compared with those without (9–11). However, those with more advanced COPD have been found not to have a significant reduction in lung cancer–specific mortality from LCS (9).

Age and smoking history are the strongest predictors of the development of lung cancer and COPD, enabling identification of a common population in which to carry out “case finding” for the two conditions. Indeed, LCS studies have reported COPD prevalence rates as high as 38% (12), almost fourfold higher than in the general population (13). Other studies have demonstrated a significant burden of undiagnosed COPD within primary care populations (13–16), though currently “screening” for asymptomatic COPD is not recommended by the U.S. Preventative Services Task Force (USPSTF) (17). Given that symptoms, exacerbation frequency, and comorbidities are important determinants of prognosis, and rate of decline of forced expiratory volume in 1 second (FEV_1_) is not currently modifiable with pharmacotherapy (18, 19), the GOLD (Global Initiative for Obstructive Lung Disease) guidance emphasizes the importance of the former parameters over spirometry values for guiding treatment decisions (20). Furthermore, comorbidities, such as CHD, hypertension, and osteoporosis, are frequent in COPD, and may be undertreated (18, 21). Optimization of these conditions may positively impact COPD outcomes. The GOLD 2019 report has recommended active COPD case finding for those with symptoms and/or risk factors (20), but it is unknown whether detecting airflow obstruction and emphysema in the LCS population confers any clinical benefit.

In this study, we aimed to explore the clinical significance of COPD and emphysema detected at LCS. Specifically, to: *1*) assess the prevalence of airflow limitation, respiratory symptoms, and radiological emphysema in an LCS population; *2*) determine whether LCS participants with COPD differ from those with “undiagnosed COPD” in terms of symptoms, comorbidities, and inhaler use, and whether symptom prevalence differs in participants with and without emphysema; and *3*) explore associations between airflow limitation, emphysema, and respiratory symptoms.

## Methods

### Study Design, Participants, and Setting

This was a nested, cross-sectional study using data from the UK-based randomized controlled LSUT (Lung Screen Uptake Trial), the design of which has been described previously (22). Briefly, individuals between 60 and 75 years of age, who were coded as current smokers within the past 5–7 years, were invited by their general practitioner for a “lung health check” (LHC) at one of two London hospitals. The primary aim of LSUT was to test differences in uptake of LCS between individuals randomly allocated to either “standard” or “targeted” invitation materials, which were designed to engage smokers from low-socioeconomic communities. Individuals attending the LHC were invited to participate in the study.

At the time of recruitment, emerging data supported that risk-based selection into screening may be preferable to the USPSTF-advocated age and smoking-based criteria (23, 24), due to enrichment of lung cancer in the selected population and a resultant optimized risk–benefit balance from LCS. In light of this evidence, both strategies were considered appropriate for selecting LCS participants. In the present study, those meeting the USPSTF criteria for LCS (i.e., ≥30 pack-years and quit ≤15 yr ago) (25), or a lung cancer risk of ≥1.51% in 6 years as determined by the Prostate Lung Colorectal Ovarian model (26) or ≥2.5% in 5 years as determined by the Liverpool Lung Project model (27), were offered LDCT. Exclusion criteria included lack of capacity to consent, physical status contraindicating LDCT, or a prior chest CT scan within the previous 12 months.

### Data Collection

Self-reported demographics (age, sex, ethnicity, education level, and Index of Multiple Deprivation [IMD] score and rank), smoking, family and medical history, height, weight, and blood pressure were collected by a study practitioner at the LHC.

#### Symptoms

Participants were asked about current or recent history of respiratory symptoms. Study practitioners were instructed to read out and explain the symptoms and ask participants to state if they experienced these symptoms currently or in the previous 12 months. Specifically, participants were asked about dyspnea (explained as “shortness of breath”), persistent cough (“a cough lasting ≥6 wk”), and lower respiratory tract infection (“a chest infection with productive cough”). Participants who reported a history of persistent cough or dyspnea within the last 12 months were regarded as having respiratory symptoms. This composite outcome was used to try to capture those participants who, in the context of airflow obstruction, may have COPD, and may reasonably be expected to undergo further medical assessment to determine this. The modified Medical Research Council dyspnea score was also assessed and recorded by the study practitioner.

#### Comorbidities

Participants were asked about a known history of COPD, asthma, previous pneumonia, CHD, hypertension (including those controlled with antihypertensives), hypercholesterolemia, diabetes mellitus, and osteoporosis. Participants were labeled as “undiagnosed COPD” if they had evidence of airflow limitation and did not report a prior diagnosis of COPD, chronic bronchitis, or emphysema. Participants were also asked about inhaled therapy use and were given names of different classes of inhalers and commonly used examples.

#### Spirometry

Spirometry was measured in all participants using a Vitalograph Micro handheld spirometer during the LHC in accordance with the joint European Respiratory Society and American Thoracic Society (28) and British Thoracic Society recommendations (29). Additional bronchodilator was not given. As timing of usage was not confirmed, we perceived the spirometry measured at the LHC to be “prebronchodilator,” as this would be the case for the majority of participants. Participant ethnicity, age, and height were used to calculate predicted values, enabling absolute and percent predicted values to be recorded. Airflow limitation was classified in accordance with GOLD (20), meaning participants were defined as having COPD if FEV_1_:forced vital capacity (FVC) was <70%. Participants with COPD with FEV_1_ ≥80%, <80% but ≥50%, <50% but ≥30%, or <30% were classified as GOLD class I, II, III, or IV, respectively.

#### Emphysema

The scans were acquired using a standard low-dose protocol, and were single read by consultant thoracic radiologists with CT reporting expertise, and experience ranging from 5 to 28 years. Visual, semiquantitative assessment of emphysema grade, based on published precedent in COPD (30), was subjectively determined on a visual scale of none, mild, moderate or severe. A total of 5% of scans was second read by a second external radiologist for quality assurance purposes.

### Sample Size and Statistical Analysis

The sample size of the LSUT cohort was based on the primary behavioral research question, and has been described in the published protocol (22). The present analysis was prespecified in the main study protocol. Participants who had never smoked or who had missing spirometry data were excluded. For the present study, 986 participants were divided into three groups: “no COPD” (FEV_1_:FVC ≥70%), “undiagnosed COPD” (FEV_1_:FVC<70% and no reported history of COPD), and “known COPD” (FEV_1_:FVC <70% with reported history of COPD).

Descriptive statistics were used to describe the demographic and clinical characteristics of participants and the prevalence of airflow limitation, emphysema, and symptoms in each group. Chi-square tests were used to determine group differences in symptom prevalence, comorbidities and inhaler use, and differences in symptom prevalence between those with and without emphysema. Univariable and multivariable logistic regression was used to explore associations between symptom prevalence, airflow limitation, and emphysema. Multivariable models were adjusted for age, smoking pack-years, reported history of osteoporosis and CHD, FEV_1_ % predicted, and grade of emphysema. Variables included in the final model were chosen if acknowledged to be *a priori* confounders for respiratory symptom prevalence. Sex, IMD quintile, and body mass index were not included in the final model, as they were believed not to confound the dependent outcome. The final model included only those who had completed an LDCT (*n* = 761). Due to the variability of current symptom prevalence within the composite outcome variable, a sensitivity analysis was performed testing the final model against an alternative outcome variable (current cough or breathlessness). Radiologist interobserver agreement was assessed using the weighted κ test (κ_w_) with quadratic weights in the 5% of LDCT scans that were double read as part of the quality-assurance process. Missing values were excluded from the analyses (and were present for only one variable: IMD score).

## Results

Of the 1,005 participants recruited to LSUT between November 2015 and July 2017, 19 were excluded because they had never smoked or due to missing data, and 986 participants were included in the present analysis. The majority of participants were white, had left school at or before the age of 15 years, were from the two most deprived IMD quintiles, and were current smokers with a heavy smoking history (Table 1).

**Table 1.** Participant characteristics by chronic obstructive pulmonary disease group

Variables	COPD Groups [*n* (*%*)] or [*median (IQR)*]		
	No COPD (*n* = *426*)	Undiagnosed COPD (*n* = *377*)	Known COPD (*n* = *183*)
Age, yr	65 (62,68)	66 (63,69)	66 (63–70)
Female	213 (50.0)	142 (37.7)	93 (50.8)
Ethnicity			
White	324 (76.1)	320 (84.9)	171 (93.4)
Black/African/Caribbean	62 (14.5)	31 (8.2)	7 (3.8)
Other	40 (9.2)	24 (6.3)	5 (2.7)
Highest level of education			
Left school at or before age 15 yr	207 (48.6)	187 (49.6)	121 (66.1)
Completed high school level or equivalent	45 (10.6)	41 (10.9)	18 (9.8)
A-levels or equivalent	44 (10.3)	41 (10.9)	11 (6.0)
Further education	23 (5.4)	15 (4.0)	9 (4.9)
Bachelor’s degree	51 (12.0)	53 (14.1)	15 (8.2)
Further higher degree	46 (10.8)	36 (9.6)	4 (2.2)
Other/prefers to say	10 (2.4)	4 (1.1)	5 (2.7)
Index of multiple deprivation quintile			
1 (most deprived)	239 (56.1)	199 (52.8)	99 (54.1)
2	136 (31.9)	141 (37.4)	60 (32.8)
3	12 (2.8)	6 (1.6)	2 (1.1)
4	1 (0.23)	1 (0.3)	0 (0)
5 (least deprived)	0 (0)	0 (0)	0 (0)
Missing	38 (8.9)	30 (12.0)	22 (12.02)
Smoking history			
Current	288 (67.6)	252 (66.8)	121 (66.1)
Former smoker	138 (32.3)	125 (33.2)	62 (33.9)
Years smoked, yr	46 (41–50)	47 (42–52)	48 (45–54)
Average smoking intensity, cigarettes/d	15 (10–20)	15 (10–20)	20 (15–25)
Lung function			
FEV_1_, L	2.29 (1.86–2.72)	2.03 (1.64–2.54)	1.57 (1.18–1.95)
FEV_1_ % predicted	91 (79–103)	79.0 (65–91)	65 (50–80)
FVC, L	2.97 (2.39–3.36)	3.33 (2.68–4.01)	2.75 (2.18–3.48)
FVC % predicted	94 (81–107)	100 (85–115)	91 (73–108)
FEV_1_:FVC, %	76.5 (73–80)	64 (59–68)	58 (50–64)
BMI, kg/m^2^	26.8 (23.6–30.1)	25.2 (22.5–28.6)	25.6 (22–29.1)
mMRC dyspnea score			
0-Breathless on strenuous exercise only	310 (72.7)	254 (67.4)	68 (37.2)
1-Slightly breathless, e.g., up hills	103 (24.2)	110 (29.2)	88 (48.1)
2-Slower than contemporaries	11 (2.6)	9 (2.4)	21 (11.5)
3-100 m exercise tolerance	1 (0.2)	4 (1.1)	6 (3.3)
4-Housebound	1 (0.2)	0 (0)	0 (0)
LDCT performed	317 (74.4)	297 (78.8)	147 (80.3)

*Definition of abbreviations*: BMI = body mass index; COPD = chronic obstructive pulmonary disease; FEV_1_ = forced expiratory volume in 1 second; FVC = forced vital capacity; IQR = interquartile range; LDCT = low-dose computed tomography; LHC = lung health check; mMRC = modified Medical Research Council Dyspnea scale.

COPD groups based on prebronchodilator spirometry at LHC appointment pre-LDCT: “no COPD” (FEV_1_:FVC at LHC ≥70%); “undiagnosed COPD” (FEV_1_:FVC at LHC <70% and no reported history of COPD); and “known COPD” (FEV_1_:FVC at LHC <70% and a reported history of COPD). Percent totals may not sum up due to rounding.

**Figure 1.**
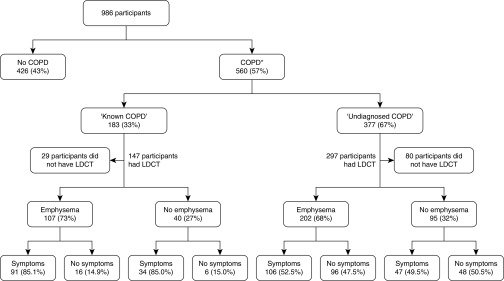
Prevalence of respiratory symptoms (inclusive of those with a history of persistent cough or dyspnea during the 12 months preceding the lung health check [LHC]) in participants with a FEV_1_:FVC <70% on the pre–low-dose computed tomography (LDCT), prebronchodilator spirometry, and with or without emphysema detected at LDCT. *Termed “chronic obstructive pulmonary disease” (COPD) solely on the basis of LHC spirometry. FEV_1_ = forced expiratory volume in 1 second; FVC = forced vital capacity.

A total of 560 (57%) participants had prebronchodilator spirometry consistent with COPD. Of those, 183 (33%) reported a prior history of COPD, whereas 377 (67%) did not, and were thus labeled “undiagnosed COPD.” The proportion of participants noted to have emphysema on their LDCT was similar in those with known (*n* = 107, 73%) compared with “undiagnosed” COPD (*n* = 202, 68%); 135 (45%) of those without emphysema had prebronchodilator spirometry consistent with COPD. Symptoms were prevalent in 91 (85%) of those with known COPD compared with approximately half of those with “undiagnosed COPD,” irrespective of the presence of emphysema (Figure 1). A total of 190 of 377 participants (50.4%) with previously unknown airflow obstruction had persistent cough or dyspnea within the last 12 months (and this amounted to 19% of the entire cohort).

**Figure 2.**
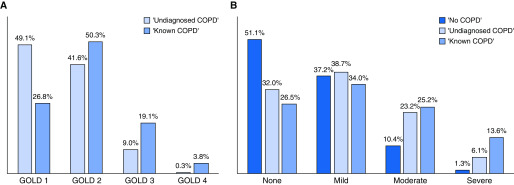
Prevalence and grade of (*A*) airflow obstruction and (*B*) emphysema by chronic obstructive pulmonary disease (COPD) group. COPD groups: “no COPD” (FEV_1_:FVC ≥70%); “undiagnosed COPD” (FEV_1_:FVC <70% and no reported history of COPD); and “known COPD” (FEV_1_:FVC <70% and a reported history of COPD). FEV_1_ = forced expiratory volume in 1 second; FVC = forced vital capacity; GOLD = Global Initiative for Obstructive Lung Diseases.

Totals of 342 (91%) and 141 (77%) participants had prebronchodilator spirometry consistent with GOLD class I or II in the “undiagnosed COPD” and known COPD groups, respectively (Figure 2A). Emphysema was prevalent in between one-half and three-quarters of participants, varying by COPD group (Figure 2B).

**Figure 3.**
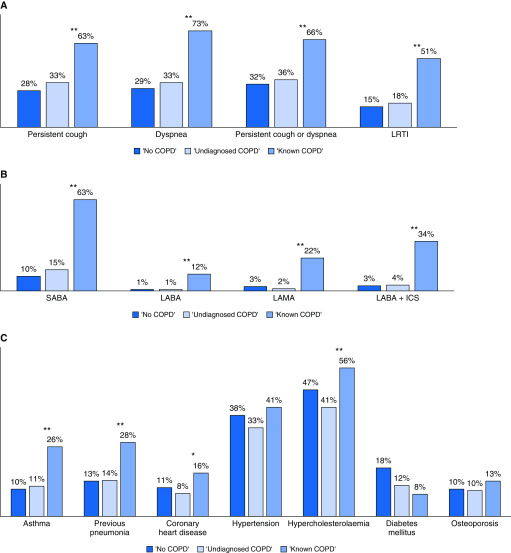
Prevalence of (*A*) reported respiratory symptoms within 12 months preceding the lung health check, (*B*) reported inhaler use, and (*C*) reported comorbidities, by chronic obstructive pulmonary disease (COPD) group. *P ≤ 0.05 and **P ≤ 0.001. COPD groups: “no COPD” (FEV_1_:FVC ≥70%); “undiagnosed COPD” (FEV_1_:FVC <70% and no reported history of COPD); and “known COPD” (FEV_1_:FVC <70% and a reported history of COPD). FEV_1_ = forced expiratory volume in 1 second; FVC = forced vital capacity; ICS = inhaled corticosteroid; LABA = long-acting β-agonist; LAMA = long-acting antimuscarinic agent; LRTI = lower respiratory tract infection; SABA = short-acting β-agonist.

The prevalence of respiratory symptoms varied from 15% to 73% by symptom and group, whereas inhaler use varied from 10% to 63% by group for short-acting β-agonists. The prevalence of both symptom and inhaler use was significantly higher in those with known COPD compared with those with undiagnosed COPD (*P* < 0.001 for all comparisons; Figure 3A and 3B). Most comorbidities were similarly distributed across groups, whereas others were significantly more common in those with known COPD compared with undiagnosed COPD, including asthma (48 [26%] vs. 43 [11%], *P* < 0.001), prior pneumonia (56 [31%] vs. 51 [14%], *P* < 0.001), hypercholesterolemia (102 [56%] vs. 154 [41%], *P* < 0.001), and CHD (30 [16%] vs. 32 [8%], *P* = 0.005) (Figure 3E).

**Figure 4.**
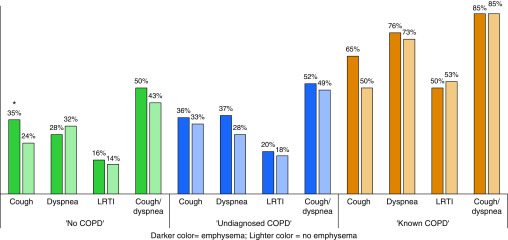
Prevalence of reported respiratory symptoms within 12 months preceding the lung health check in those with and without emphysema by chronic obstructive pulmonary disease (COPD) group. COPD groups: “no COPD” (FEV_1_:FVC ≥70%); “undiagnosed COPD” (FEV_1_:FVC <70% and no reported history of COPD); and “known COPD” (FEV_1_:FVC <70% and a reported history of COPD). *P ≤ 0.05. FEV_1_ = forced expiratory volume in 1 second; FVC = forced vital capacity; LRTI = lower respiratory tract infection.

Respiratory symptoms did not vary by presence or absence of emphysema on LDCT. The only statistically significant difference was for persistent cough, which was higher in those with no airflow limitation, but with emphysema, on LDCT (*n* = 55, 35%) compared with those with no airflow limitation and no emphysema on LDCT (*n* = 24, 22%) (*P* = 0.03; Figure 4).

Variables not found to be significantly associated with symptom prevalence in univariable logistic regression analyses included age, sex, IMD quintile, body mass index, and smoking status. Unadjusted and adjusted (for age, pack-year smoking history, history of osteoporosis and CHD, emphysema, and airflow obstruction) odds ratios (ORs) for symptom prevalence are presented in Table 2. The association between respiratory symptoms and airflow limitation remained significant for participants with FEV_1_:FVC <70% and FEV_1_ ≥50% (adjusted OR [aOR], 1.74; confidence interval [CI], 1.57–2.17), and FEV_1_ <50% (aOR, 4.6; CI, 2.17–9.77). Conversely, the association between respiratory symptoms and grade of emphysema only retained significance for those with severe emphysema (aOR, 4.00; CI, 1.57–10.2).

**Table 2.** Adjusted and unadjusted odds ratios for presence of persistent cough or dyspnea currently or within the past 12 months with airflow obstruction and emphysema in this cohort

	Unadjusted OR (CI)	*P* Value	Adjusted OR (CI)	*P* Value
Age, yr				
60–63	1	0.32	1	0.004
64–67	0.75 (0.55–1.02)		0.54 (0.37–0.79)	
68–72	0.84 (0.59–1.19)		0.55 (0.35–0.84)	
73–76	0.84 (0.55–1.29)		0.53 (0.31–0.88)	
Pack-year smoking history				
0–20	1	<0.001	1	<0.001
21–40	1.54 (1.09–2.15)		1.13 (0.73–1.76)	
41–60	2.02 (1.42–2.88)		1.58 (1.00–2.49)	
>60	4.64 (2.94–7.31)		3.28 (1.87–5.77)	
History of osteoporosis				
No	1	0.045	1	0.021
Yes	1.53 (1.00–2.33)		1.82 (1.08–3.07)	
History of CHD				
Yes	1	<0.001	1	0.019
No	2.17 (1.41–3.35)		1.85 (1.09–3.13)	
Airflow obstruction				
FEV_1_:FVC ≥70%	1	<0.001	1	<0.001
FEV_1_:FVC <70% & FEV_1_ >50%	1.74 (1.34–2.27)		1.57 (1.14–2.17)	
FEV_1_:FVC <70% & FEV_1_ <50%	5.59 (3.03–10.3)		4.60 (2.17–9.77)	
Emphysema				
None	1	<0.001	1	0.011
Mild	1.12 (0.81–1.55)		1.05 (0.74–1.48)	
Moderate	1.64 (1.09–2.48)		1.33 (0.85–2.09)	
Severe	5.88 (2.41–14.4)		4.00 (1.57–10.2)	

*Definition of abbreviations*: CHD = coronary heart disease; CI = confidence interval; FEV_1_ = forced expiratory volume in 1 second; FVC = forced vital capacity; OR = odds ratio.

Values given to two significant figures.

In the sensitivity analysis, the findings were not significantly altered using current cough or breathlessness as the outcome variable (data not shown). Radiologist interobserver agreement in the 5% of LDCT scans that were double read as part of the quality assurance process was found to be “good” according to Landis and Koch (31) (κ_w_ = 0.65, *P* < 0.001).

## Discussion

In this cross-sectional cohort study of 986 individuals attending an LHC, 57% had prebronchodilator spirometry consistent with COPD, 67% did not report a prior history of COPD (thus labeled “undiagnosed COPD”), and 32% of those with “undiagnosed COPD” had no evidence of emphysema on LDCT. Prevalence of symptoms, inhaler use, and certain comorbidities were significantly higher in those with known compared with undiagnosed COPD. The presence of airflow obstruction was more strongly associated with respiratory symptoms than emphysema. These results suggest that emphysema alone does not robustly identify participants with COPD.

The proportion of participants with spirometry consistent with COPD is higher in the present study (57%) than reported in NLST participants (10) (34%). Emphysema prevalence was higher than reported in other cohorts (32). These findings may be explained by the population recruited to this “real-world” study, where participants were invited for an National Health Service LHC rather than a clinical trial. The present study participants were older, more deprived, less educated, and more likely to be currently smoking when compared with NLST participants (6).

Whether case finding of individuals with airflow limitation or emphysema in the absence of respiratory symptoms is of value is controversial. Studies have reported increased exacerbation frequency in asymptomatic individuals with airflow limitation (13) and with radiological findings consistent with COPD (33). Few studies have evaluated the use of pharmacotherapy in asymptomatic COPD. One study in China demonstrated an improvement in annual post-bronchodilator FEV_1_ decline with tiotropium compared with placebo in patients with mild COPD (34), signaling possible benefit with pharmacotherapy for mild COPD, though more studies are needed. Presently, for those with minimal symptoms, simply lifestyle modifications, including smoking cessation, are recommended (18, 35).

We sought to explore what proportion of participants had “clinically significant” COPD (i.e., undiagnosed airflow obstruction plus respiratory symptoms). To our knowledge, no prior studies have reported COPD symptom prevalence in LCS participants, though findings consistent with ours have been reported in smokers without demonstrable airflow limitation (15). This group was previously termed “GOLD 0,” and although studies in this group are ongoing (36); this term is not used clinically, as it does not help predict who may later develop airflow obstruction (37). Our estimate that up to 19% of participants had potentially clinically significant undiagnosed COPD is an important consideration when devising LCS protocols and infrastructure.

The finding that those with and without emphysema were similar in terms of respiratory symptoms was surprising, as an association between all-cause mortality in smokers without airflow limitation and with emphysematous changes has been reported (38). This association, which was confirmed in the multivariable analysis, suggests that airflow limitation may be more pertinent for detecting “clinically significant” disease than LDCT-detected emphysema. Thus, measurement of spirometry in the LCS setting could enhance prognostication and education, all of which are not adequately achieved using the presence of emphysema on CT alone.

The integration of spirometry with symptom assessment in the LCS setting could enhance early detection of COPD and provide an opportunity to implement evidence-based smoking cessation interventions. Visual feedback of medical imaging may promote behavior change, such as smoking cessation (39), and research is underway to determine whether personalized feedback of LDCT-detected emphysema are effective at enhancing smoking cessation (40). It could also enable promotion of vaccination against respiratory infection, attention to diet, activity, symptom awareness, and initiation of appropriate pharmacotherapy. This needs to be balanced against risks associated with medication overuse, cost, primary care resource utilization, and impact on insurance eligibility. An added consideration is that a diagnosis of COPD may influence the overall benefit that an individual may stand to gain from LCS. It has been noted that having undiagnosed or early COPD confers a greater relative risk reduction in lung cancer–specific mortality than observed in NLST, whereas having more advanced COPD may be associated with no mortality benefit (9). As such, the shared decision-making discussion would benefit from spirometry readings to determine an individual’s specific potential benefits and harms of screening. Such issues have been highlighted in a recent official American Thoracic Society statement (41) as important factors to be raised in the LCS shared decision consultation.

The study was limited by possible selection bias, as it is possible that individuals with symptoms were more likely to attend an LHC appointment. The sample was predominantly of a white ethnic background, with a high current smoking rate and from a more socioeconomically deprived background than previous studies, and therefore likely to have higher rates of COPD and lung cancer than the wider LCS-eligible population.

Nevertheless, similar lung cancer prevalence and demographics have been described in other U.K. cohorts (42, 43), and, in light of emerging evidence advocating selection of LCS-eligible individuals based on lung cancer risk (23, 44), the findings reported here are generalizable to the desired population. We did not collect data on exacerbation history, which would enhance our classification of COPD severity. Many of the outcome measures were dependent on self-reported history and subject to recall bias. We acknowledge that some participants may have underreported a diagnosis of COPD or misreported COPD as asthma. We did not complete post-bronchodilator spirometry to classify COPD; several other studies, including NLST (10), have employed spirometry without additional bronchodilation as a metric, and GOLD has accepted this as a pragmatic approach to COPD case finding, directing people for additional testing to confirm the diagnosis (20). The present analysis could not account for unmeasured factors affecting symptom perception and prevalence, such as psychosocial influences and premorbid fitness. Emphysema was graded visually rather than quantitatively, and is subject to some interobserver bias, though “good” agreement between observers was detected on the 5% of scans read by two radiologists for quality assurance purposes. Despite these limitations, the findings presented here are novel with respect to the evaluation of associations between symptomatology, radiology, and physiological parameters in COPD, and highlight the potential benefits arising from incorporating spirometry into the assessment for LCS and the LHC approach adopted in LSUT.

### Conclusions

This study has demonstrated the significant burden of COPD, respiratory symptoms, and comorbidities in a cohort of individuals attending an LHC. Half of those with undiagnosed COPD had respiratory symptoms, whereas one-third did not have emphysema. Airflow limitation was a stronger predictor for respiratory symptoms than emphysema. This emphasizes the importance and value of spirometry at LCS over and above detecting emphysema on LDCT. Further studies should evaluate the impact of early detection of airflow limitation and emphysema through LCS on behavior change and long-term COPD outcomes. To this end, we are expanding our studies to a larger cohort within the SUMMIT study (45).

## Supplementary Material

Supplements

Author disclosures
