# Supplementary material for: Prevalence, Symptom Burden, and Underdiagnosis of Chronic Obstructive Pulmonary Disease in a Lung Cancer Screening Cohort
Source: Ann Am Thorac Soc. 2020 Jul;17(7):869–78. doi: 10.1513/AnnalsATS.201911-857OC (PMC7328177; doi:10.1513/AnnalsATS.201911-857OC)
Supplement: Supplements [file AnnalsATS.201911-857OC.html]

Prevalence, Symptom Burden, and Underdiagnosis of Chronic Obstructive Pulmonary Disease in a Lung Cancer Screening Cohort | Annals of the American Thoracic Society

- disclosures.pdf (474 KB)
